# Consent to Specimen Storage and Continuing Studies by Race and Ethnicity: A Large Dataset Analysis Using the 2011-2012 National Health and Nutrition Examination Survey

**DOI:** 10.1155/2014/120891

**Published:** 2014-11-18

**Authors:** Andre Gabriel, Catherine Crawford Cohen, Carolyn Sun

**Affiliations:** ^1^Department of Medicine, Division of Pulmonary, Allergy, & Critical Care Medicine, Columbia University Medical Center, 622 West 168 Street, New York, NY 10032, USA; ^2^Center for Health Policy, Columbia University School of Nursing, 630 West 168th Street, New York, NY 10032, USA; ^3^Columbia University School of Nursing, 630 West 168th Street, New York, NY 10032, USA

## Abstract

*Purpose*. To determine if significant differences exist in consent rates for biospecimen storage and continuing studies between non-Hispanic Whites and minority ethnic groups in the National Health and Nutrition Examination Survey (NHANES). *Methods*. Using logistic regression, we analyzed 2011-2012 NHANES data to determine whether race/ethnicity, age, gender, and education level influence consent to specimen storage or future testing. *Results*. Compared to non-Hispanic Whites, some minorities were less willing to donate a specimen for storage and continuing studies, including other Hispanics (non-Mexican) (OR 0.236, 95% CI: 0.079, 0.706), non-Hispanic Asians (OR 0.212, 95% CI: 0.074, 0.602), and other/multiracial ethnic groups (OR 0.189, 95% CI: 0.037, 0.957). Within race and ethnic groups, those aged 20–39 years (OR 2.215, 95% CI: 1.006–4.879) and 40–59 years (OR 9.375, 95% CI: 2.163–40.637) are more willing than those over 60 years to provide consent. *Conclusion*. Lower consent rates by other Hispanics, non-Hispanic Asians, and other/multiracial individuals in this study represent the first published comparison of consent rates among these groups to our knowledge. To best meet the health care needs of this segment of the population and to aid in designing future genetic studies, reassessment of ethnic minority groups concerning these issues is important.

## 1. Introduction

It is well recognized [1] that Latino Americans and African-Americans carry a disproportionate burden of chronic disease in the US [2], including higher death rates associated with these diseases among Blacks as compared to non-Hispanic White Americans [2, 3]. Despite these persistent racial and ethnic health disparities, ethnic minority groups remain underrepresented in research intended to reduce disease burdens [3]. Given the current focus on personalized medicine [4], medical research increasingly requires biological samples from research participants [5] which necessitates extensive resources to collect a diversity of relevant genotypes [6]. Those who choose to consent to biospecimen storage and continuing studies may be overrepresented in ongoing and future research [3, 6]. Therefore, understanding which groups are most likely to consent for specimen storage and continuing research is important to interpret results of the studies using these specimens.

Previous studies demonstrate that racial and ethnic groups are not equally willing to give biological specimens. Lower participation rates among ethnic minorities were attributed to concerns regarding exploitation by medical researchers, discrimination, confidentiality, inequities between those benefiting from the research and those participating in research, and a lack of direct benefit from the research or disinterest in genetic research [3]. Some of these publications examined the National Health and Nutrition Examination Survey (NHANES) data collected from 1999 to 2008 [7–9], a large dataset. Data collection for NHANES includes a medical examination interview conducted in English, Spanish, mandarin Chinese (both traditional and simplified), Korean, and Vietnamese [10]. Hence, observed disparities among ethnic groups likely extend beyond language barriers and can provide information about health and participation willingness across these groups.

Findings of previous studies regarding consent rates by racial and ethnic groups may not be relevant due to key changes in NHANES since 2007. First, participants are no longer given a separate consent form for DNA storage for future use. Consent rates increased overall after this new format was implemented. Second, the consent forms for collection of samples included more detailed information regarding genetic testing [9]. Third, NHANES sampling was also adjusted to oversample all Hispanics, rather than only Mexican-Americans, as had been done previously [11]. Fourth and most importantly, self-reported ethnicity data was changed to include Mexican-American, other Hispanics ethnicity (those who are self-identified as Hispanic but not Mexican-American), and Asian options for the first time in the 2011-2012 dataset [10]. Despite these key changes in consent forms, sampling strategy, demographic categories, to our knowledge, NHANES consent rates by race and ethnicity have not been reexamined and compared.

This study aims to determine if there is a difference between minority racial and ethnic groups and Whites in their consent rates for biological specimen storage and continuing research that required a biological specimen among adults who participated in the 2011-2012 NHANES cycle. Analysis of these data may provide new information about the relative willingness of minority ethnic groups to consent to biospecimen storage and donation for continuing studies [12]. This study may also identify potential differences in consent rates within the Hispanic population, which comprises the third largest ethnic minority population and second fastest growing population in the US [13].

## 2. Materials and Methods

### 2.1. Sources of Data

The NHANES is a national, ongoing study by the Centers for Disease Control and Prevention (CDC) that is designed to assess the health and nutrition status of residents of the US. This study includes a survey and physical examination information that are collected both in the home and at mobile medical examination centers, respectively [14]. The NHANES uses a sample that is statistically determined* a priori* to give a nationally representative sample. This is a complex, multistage probability sampling design of noninstitutionalized civilians within the 50 states. First a sample is gathered within a county, then within a segment of the county, then households within the segment, and finally individuals within the household [15, 16]. A weighting scheme is used to give a representative proportion to certain population subgroups of special interest. This increases the reliability and precision of estimates of health status indicators for these populations [14, 15]. To be included, participants must live in the US. Certain populations (e.g., the elderly, non-Hispanic Blacks, and Hispanics) are purposefully oversampled to provide a representative population. US citizens that are incarcerated, institutionalized, or working in the military are not eligible [14].

**Figure 1:**
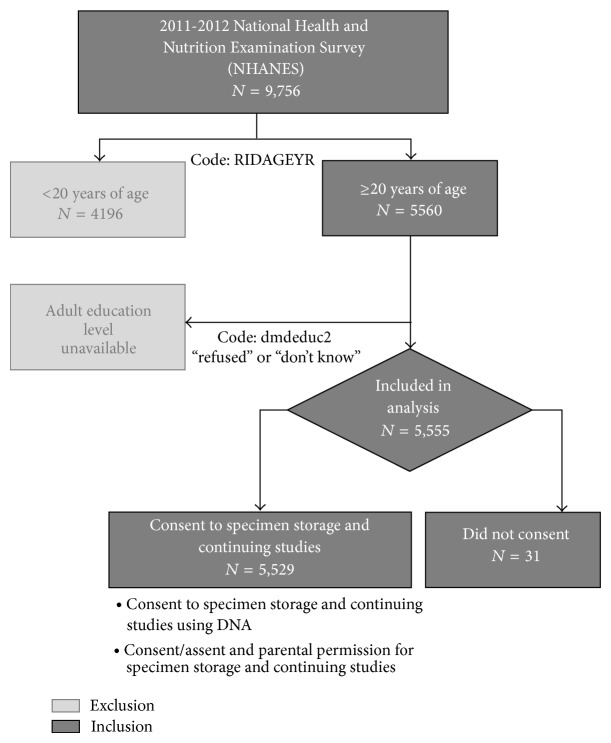
Participant selection process for data analysis, 2011-2012 NHANES.

In this study, only participants aged 20 years and older were included in our analyses since (1) NHANES collected education data on those aged 20 and older [10] and (2) consent for participation in “Specimen Storage and Continuing Studies Using DNA” was only offered to those over 20 years old [17]. Further, participants with missing data for any variable of interest were not included (see Figure 1).

### 2.2. Variables

The dependent variable was consent on either one of two consent forms regarding biospecimen storage and continuing studies (additional to the NHANES and exam consent) [17, 18]. Information on consent status is not publically available because the specimen IDs are linked to the sequence numbers in a separate database to maintain confidentiality. To obtain consent data, the authors provided a list of sequence numbers for all participants from the 2011-2012 survey cycles to the CDC where they were then matched with consent status by the CDCs information management programmer.

Independent variables were demographic information regarding study participants: race/ethnicity, age, gender, educational level, and income poverty ratio, as obtained from the 2011-2012 NHANES dataset that is available online. Categorical groups for race and age were defined in accordance with the groups created by the CDC, and both were self-reported [15]. Prior studies have discrepant results regarding the influence of age on consent to biospecimen donation. To provide an appropriate comparison to studies which have examined the relationship between age and consent [7–9, 19–25], we categorized age in the same manner. In the analyses of the NHANES data collected from 1999 to 2004, sex differences in consent rates varied across intervals [8]. Therefore, influence of sex on consent was included. Regarding education level, previous studies found high school degree or less were more likely to donate (91.6%, CI: 90.3–92.9) versus some college reference group (89.1%, CI: 87.9–90.4) [8] or donate after second request (4.36, 95 percent CI: 1.33, 14.27), and those with some college or college graduates were less likely to consent compared to those with less than a high school education (odds of consenting OR 0.49 CI: 0.27, 0.86 *P* = 0.014) [21]. In contrast, McDonald et al. found that respondents with some college education and college graduates were more likely to donate a blood or saliva sample compared with those with less education (OR 1.60 CI: 0.81, 3.14, *P* = 0.18) [26]. Therefore, educational attainment was categorized as in previous studies. Existing evidence on the role of income on participant willingness to donate a specimen is mixed [7–9, 23]. However, the study by McQuillan et al. found that in NHANES (2001-2002), higher incomes were less likely to consent to future research including genetic studies [8]. Therefore, as per their analysis, income was included as income poverty ratio.

Since unidentifiable, publicly available data were used for this analysis, conditions for exemption from Institutional Review Board (IRB) review have been met in accordance with the National Institutes of Health, Office of Extramural Research (NIH/OEP) Regulations on Human Subjects Protection and Inclusion [27].

### 2.3. Data Analysis

To determine the relationship between consent and race/ethnicity, we used SAS 9.3 statistical software [28] to run a logistic regression using consent for genetic research as the dependent variable with education level, gender, income poverty ratio, and ethnicity as the independent variables, using NHANES weighting according to guidelines [15, 29].

A forward selection approach was followed in the model building process. Independent variables significantly associated with the outcome (*P*-value of < 0.25) were selected for the initial model. A variable was included in the final model if the Wald chi-square test statistic showed a level of significance of *P* < 0.05 and the regression coefficients changed significantly when it was removed from the multiple logistic regression model. We then checked for paired interactions among the variables using the Wald chi-square test and likelihood ratios. Any significant interaction term was included in the final model. To test for goodness of fit in our model, we obtained the Hosmer-Lemeshow test statistic using the lackfit feature on SAS software. We used an alpha of 0.05 and the Wald test to generate confidence intervals at the 95% significance level for the ORs.

## 3. Results

Of the 9,756 NHANES participants 2011-2012, 5,560 individuals over 20 years old were included in our analysis. After those who responded “refused” (*n* = 2) or “don't know” (*n* = 3) to education level were excluded, 5,555 individuals were included in the analysis. The included sample was 49.3% male and 9.7% Mexican-American, 10.4% other Hispanics, 36.7% non-Hispanic White, 26.2% non-Hispanic Black, 14.3% non-Hispanic Asian, and 2.7% multiracial/other races. Of the included individuals, 99.4% consented to give a biospecimen for future research.  Table 1 outlines the characteristics of included NHANES participants along all variables of interest.

**Figure 2:**
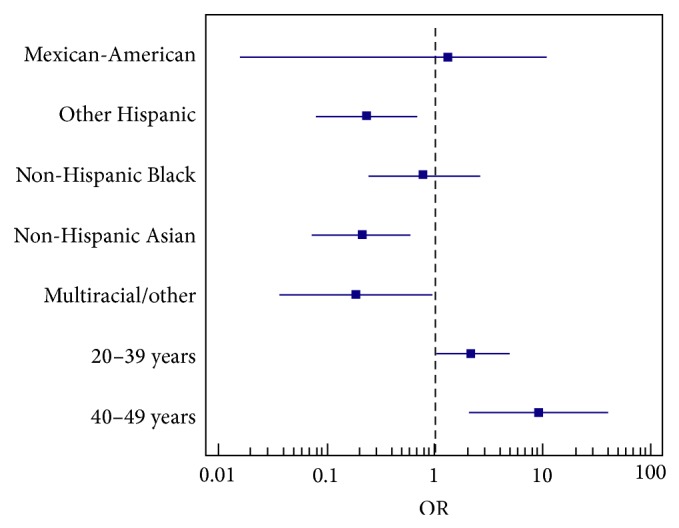
Odds ratios and 95% CI of each predictor of consent.

Age group, gender, education, and race/ethnicity were each individually significantly associated with consent to give a biospecimen in simple logistic regressions (*P* < 0.25, see Table 2 and Figure 2). There were no interactions between the variables of interest across the categories. Age group and race/ethnicity were found to be significantly associated with consent (*P* < 0.05, data not shown).

Controlling for age group, other Hispanics were statistically less willing to donate a specimen (OR 0.236, 95% CI: 0.079, 0.706), as were Non-Hispanic Asians (OR 0.212, 95% CI: 0.074, 0.602). Subjects self-identified as other/multiracial were also statistically less likely to consent (OR 0.189, 95% CI: 0.037, 0.957). These findings indicate that the odds of other Hispanics consenting to biospecimen research were 0.24 times the odds of non-Hispanic Whites given the same age group.

In this analysis, among race/ethnic groups, those in the 20–39 years and 40–59 years age groups are more willing than those in the over 60 years age group to provide consent for storage of a biospecimen for use in future studies with ORs of 2.215 (95% CI: 1.006–4.879) and 9.375 (95% CI: 2.163, 40.637), respectively.

## 4. Discussion

Given that the 2011-2012 NHANES dataset is the first to include the category of non-Hispanic Asians for race/ethnicity, the lower consent rates by non-Mexican Hispanics, non-Hispanic Asians, and other/multiracial individuals are consistent with previous findings that some ethnic minority groups may be less willing to consent to provide a biospecimen [2, 7–9, 19–23, 25, 30] while adding depth to these results through the introduction of more specific ethnic minority groups.

An interesting finding in our study is that non-Hispanic Blacks had consent rates on par with non-Hispanic Whites. These results were not consistent with previous studies where Blacks have had lower consent rates to specimen donation [2, 7–9, 19–23, 25]. These data may reflect aggressive recruitment of non-Hispanic Blacks or the ability of researchers within the study to build trust with recruits [31, 32], perhaps concomitantly with increasing public interest in genetic research and personalized medicine [31–33]. Additional research is warranted to confirm these findings.

In fact, there is a paucity of information on consent rates among ethnic minorities but in the few studies to date, studies have included very few minorities other than African-Americans [2, 4, 7–9, 19–23, 25]. Consent rates among non-Mexican Hispanics have not been explored. As public awareness and salience of genetic research for all US populations increases [34], it is important to reassess attitudes of ethnic minority groups concerning these issues. While minorities make up a growing proportion of the US population, there continues to be a lack of information on how to best meet the health care needs of this segment of the population [30, 35, 36]. These observations regarding different consent rates between age groups add new evidence where previous studies had conflicting results [2, 7–9, 19–23, 25].

Limitations of our study include that we were unable explore the reasons for nondonation. Future studies should include qualitative assessments of the reasons for withholding consent for storage for future studies. These may include health literacy, participant involvement in study development, and the use of community health workers for data collection and recruitment, all of which have been suggested in other studies as potential factors influencing consent [30, 36, 37]. Although we cannot explain differences in consent rates, our findings have significant implications for future studies that include biospecimen collection.

Research utilizing biospecimen holds promise for assisting in better care for a host of diseases, but results will be less generalizable without the inclusion of minorities [37]. This analysis also illuminates the fact that categorization of minority ethnic groups is important in uncovering the reasons for nonparticipation by varying subgroups. This study demonstrates that already underrepresented groups may be less willing to consent to this important research. Therefore, it provides rationale for future studies to understand the reasons for nonparticipation and consequently, to support future research that requires biospecimens.

## Figures and Tables

**Table 1:** Consent to biospecimen research among 2012 NHANES participants aged 20 years or more by demographic categories.

Variable	*n*	%
Consent		
Yes	5529	99.4
Gender		
Female (reference)	2820	49.28
Male	2740	50.72
Education		
Less than 9th grade	550	9.89
9–11th grade	782	14.06
High school/GED or equivalent	1169	21.03
Some college or AA degree	1657	29.80
College graduate or above	1397	25.13
Refused	2	0.04
Do not Know	3	0.05
Race		
Mexican-American	540	9.7
Other Hispanics	578	10.4
Non-Hispanic White (reference)	2041	36.7
Non-Hispanic Black	1455	26.2
Non-Hispanic Asian	749	14.3
Multiracial/other race	152	2.7
Age		
20–39 years	1957	35.2
40–59 years	1812	32.6
60+ years (reference)	1791	32.2

**Table 2:** Relative odds for consent to biospecimen storage and future research in adult participants of the 2011-2012 NHANES aged 20 years or more.

Independent variables^*^	OR	95% CI	*P* value
Age (in years)			
20–39	1.977	(0.910, 4.293)	0.0851
40–59	8.647	(2.004, 37.317)	0.0038
Race^**^			
Mexican-Asian	1.589	(0.191, 13.228)	0.6683
Other Hispanics	0.210	(0.073, 0.608)	0.0040
Non-Hispanic Black	0.855	(0.260, 2.807)	0.7962
Non-Hispanic Asian	0.257	(0.091, 0.725)	0.0102
Multiracial/other races	0.221	(0.044, 1.105)	0.0660
Race overall			0.0085
Education			
<9th grade	0.518	(0.211, 1.273)	0.1518
≥high school Graduate/GED equivalent	4.463	(0.603, 33.034)	0.1430
Education overall			0.0997
Gender (female)	0.564	(0.270, 1.180)	0.1284

^*^Note: each row represents output of one simple logistic regression between consent (dependent variable) and the independent variable listed in this column.

^**^Race determined by subject self-report, as recorded in NHANES code RIDRETH3.
